# ICAM-1 mediated cell-cell adhesion exerts dual roles on human B cell differentiation and IgG production

**DOI:** 10.1016/j.isci.2023.108505

**Published:** 2023-11-22

**Authors:** Shuai Liu, Zhi-cui Liu, Mei-yu Zhang, Shu-jun Wang, Meng Pan, Ping Ji, Cheng Zhu, Ping Lin, Ying Wang

**Affiliations:** 1 Shanghai Institute of Immunology, Department of Immunology and Microbiology, Key Laboratory of Cell Differentiation and Apoptosis of Chinese Ministry of Education, Shanghai Jiao Tong University School of Medicine, Shanghai 200025, China; 2 School of Life Sciences and Biotechnology, Shanghai Jiao Tong University, Shanghai 200240, China; 3 Department of Diagnostic Laboratory, Shanghai Mental Health Center, Shanghai Jiao Tong University School of Medicine, Shanghai 200030, China; 4 Department of Dermatology, Shanghai Tenth People’s Hospital, Tongji University School of Medicine, Shanghai 200072, China; 5 Department of Dermatology, Ruijin Hospital, Shanghai Jiao Tong University School of Medicine, Shanghai 200025, China; 6 Wallace H. Coulter Department of Biomedical Engineering, Georgia Institute of Technology, Atlanta, GA 30332, USA; 7 Shanghai Institute of Virology, Shanghai Key Laboratory of Emergency Prevention, Diagnosis and Treatment of Respiratory Infectious Diseases, Shanghai 200025, China

**Keywords:** Molecular biology, Immunology, Components of the immune system, Cell biology, Cancer

## Abstract

Intercellular adhesion molecule 1 (ICAM-1) plays prominent roles in mediating cell-cell adhesion which also facilitates B cell activation and differentiation with the help from CD4^+^ T cells. Here, we have reported a unique phenomenon that increased ICAM-1 on purified human CD4^+^ T cells upon anti-CD3/CD28 stimulation enhanced CD4^+^ T-B cell adhesion whereas induced less B cell differentiation and IgG production. This was largely due to increased PD-1 expression on CD19^hi^ B cells after coculturing with hyperactivated CD4^+^ T cells. Consequently, ICAM-1 blockade during CD4^+^ T cell-B cell coculture promoted IgG production with the activation of ERK1/2 and Blimp-1/IRF4 upregulation. Consistently, CD4^+^ T cells from moderate-to-severe SLE patients with high ICAM-1 expression mediated less IgG production after T-B coculture. Therefore, ICAM-1-mediated human CD4^+^ T-B cell adhesion provides dual roles on B cell differentiation and IgG production partially depending on expression levels of PD-1 on B cells, supporting cell adhesion and subsequent PD-1 induction as an alternative intrinsic checkpoint for B cell differentiation.

## Highlights

• ICAM-1 strengthens CD4^+^ T-B cell adhesion for B cell activation and differentiation
• Tighter CD4^+^ T-B cell adhesion restrains IgG production in a PD-1/PD-L1 dependent manner
• T-B interaction is an alternative checkpoint to regulate B cell differentiation and IgG secretion

## Graphical abstract

**Figure:**
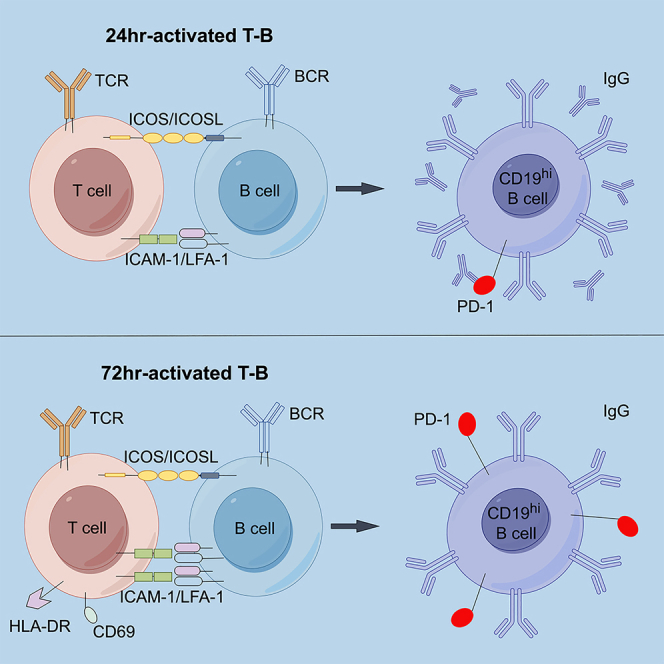

## Introduction

Intercellular adhesion molecule 1 (ICAM-1, also known as CD54) belongs to the family member of cell adhesion molecules characterized as a type I transmembrane glycoprotein. It is constitutively expressed on a variety of hematopoietic cells including B cells, dendritic cells (DCs), and follicular DCs as well as endothelial cells^1^ at low levels at the steady state. ICAM-1 interacts with integrin family member lymphocyte function-associated antigen-1 (LFA-1, CD11a/CD18) to mediate cell-cell adhesion at the early stage of immune cell activation.^2^^,^^3^ For instance, ICAM-1/LFA-1 ligation participates in the molecular machinery supporting T cell activation through promoting T cell adhesion to antigen-presenting cells (APCs) and facilitating T cell antigen receptor (TCR) recognition of major histocompatibility complex (MHC)-peptides on APCs.^4^^,^^5^^,^^6^ Upregulation of ICAM-1 after cell activation is demonstrated to be able to modulate the magnitude of immune responses.^7^^,^^8^

The roles of ICAM-1 in B cell-engaged humoral immunity have been elucidated especially in germinal centers (GCs). Within GCs, ICAM-1/LFA-1 ligation induces the signals supporting cognate interactions of B cells with follicular T helper (Tfh) cells for the generation and maintenance of humoral immunity.^9^ ICAM-1/LFA-1 interaction between T cells and B cells lowers the threshold of B cell activation depending on cytohesin-1 and Jun-activating binding protein 1 (JAB-1), which promotes T-B cell adhesion and synapse formation respectively.^4^ Furthermore, ICAM-1/2 on B cells is essential for long-lasting cognate T-B interactions and selection of low-affinity B cell clones in T cell-dependent antibody immune responses in mice.^10^ During human T-B cell collaboration, ICAM-1/LFA-1 interactions are also necessary for both the induction of human B cell proliferation and differentiation as well as the induction of IL-2 production in CD4^+^ T cells.^11^ LFA-1 on resting B cells and ICAM-1 on activated CD4^+^ T cells thus play a critical role in initial T cell-dependent B cell activation.^11^^,^^12^ The engagement of ICAM-1 in B cell activation and differentiation has also been implies from several pathogenic antibody-mediated disorders.^13^^,^^14^^,^^15^^,^^16^ For instance, in system lupus erythematosus (SLE) non-synonymous *ICAM-1* rs5498 (ICAM^Lys469Glu^) is reported to be associated with the increase in soluble ICAM-1 (sICAM-1) levels and the susceptibility to SLE.^17^ sICAM-1 is dramatically augmented in the periphery of SLE patients as well.^18^

In the present study, we have observed an unusual phenomenon that purified human CD4^+^ T cells upon anti-CD3/CD28 at different time points exert dual roles in regulating B cell differentiation and IgG production. While TCR/CD28 stimulation induced ICAM-1 upregulation in a time-dependent manner, IgG production in the supernatants of CD4^+^ T-B cell co-culture was higher when CD4^+^ T cells were stimulated for 24 h^19^^,^^20^ than those for 72 h. Moreover, ICAM-1 blockade on CD4^+^ T cells upon 24 h’ stimulation suppressed IgG production whereas that on CD4^+^ T cells upon 72 h’ stimulation promoted IgG production. The paradoxical effects of ICAM-1 on human B cell activation and IgG production with the help from activated CD4^+^ T cells for 72 h were in part due to the up-regulation of PD-1 expression on activated CD19^hi^ B cells after T-B coculture. Addition of PD-L1-Fc protein in 72 hrs-stimulated CD4^+^ T-B cell co-culture attenuated the increase in IgG production upon ICAM-1 blockade. More interestingly, when co-culturing B cells with autologous CD4^+^ T cells from moderate-to-severe SLE patients highly expressing ICAM-1, the blockade of ICAM-1 also induced increased IgG production whereas no increase was observed from mild SLE with low-expression of ICAM-1 on CD4^+^ T cells. Our data thus support that hyperactivation of CD4^+^ T cells enhances the adhesion with B cells through increased ICAM-1 expression, which is prone to promote the induction of PD-1 on B cells and exert intrinsically negative modulation on B cell differentiation and IgG production.

## Results

### IgG production has decreased when B cells are co-cultured with CD4^+^ T cells upon TCR/CD28 activation for 72 h compared to those for 24 h

**Figure 1:**
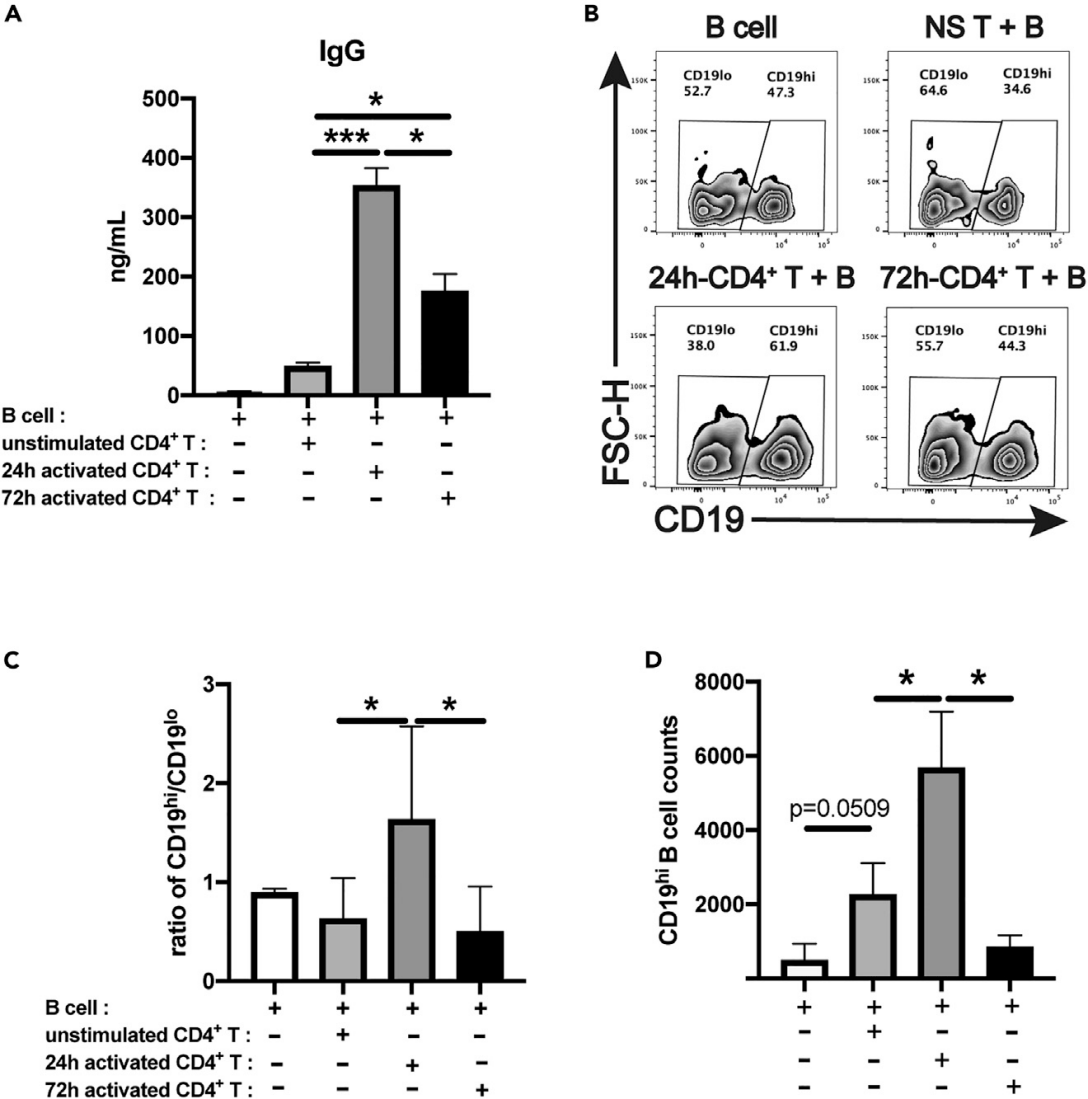
Decreased IgG content and CD19^hi^ B cell subpopulation after B cells are co-cultured with CD4^+^ T cells upon TCR/CD28 activation for 72 h when compared to those for 24 h Purified human CD4^+^ T cells from health donors were stimulated with human T-Activator CD3/CD28 Dynabeads for 24 h or 72 h. (A) IgG content in the supernatants after B cells were co-cultured with autologous activated or resting CD4^+^ T cells for 12 days (n = 10). (B) Presence of CD19^lo^ and CD19^hi^ B cell subpopulations in total B cell subset after the co-culture of resting (NS T + B) or activated CD4^+^ T cells (24h-CD4^+^T+B and 72h-CD4^+^T+B) at Day 12 where B cell alone was blank control. (C) Comparison of the ratios of CD19^hi^/CD19^lo^ B cells (n = 10) in different CD4^+^T-B cell co-culture groups. CD19^hi^/CD19^lo^ B cell ratios of the blank controls of each individual HC was used for normalization. (D) The absolute counts of CD19^hi^ B cells after CD4^+^T-B cell coculture. The data were representative of at least three independent experiments. Data were represented by mean ± SD. In Figures 1A, 1C and 1D, the Kruskal-Wallis test with subsequent Duuns multiple-comparison test was used. ∗: p < 0.05; ∗∗∗: p < 0.001.

We have previously established an *in vitro* CD4^+^ T-B cell co-culture system to investigate the roles of human CD4^+^ T cell activation in promoting autologous B cell differentiation.^19^^,^^20^ It was evident that upon anti-CD3/CD28 stimulation for 24 h, CD4^+^ T cells promoted B cell differentiation and IgG/IgM production efficiently in the *in vitro* co-culture. Similar to the previous results, we have also observed the increase in IgG levels in the supernatants of CD4^+^ T-B co-culture when CD4^+^ T cells were activated for 24 h. Unexpectedly, IgG levels were lower in the supernatants with CD4^+^ T cells upon 72h′ stimulation than those with 24 h stimulated CD4^+^ T cells whereas still higher than those with resting CD4^+^ T cells (Figure 1A).

We have also reported that CD19^hi^ B cells, an activated B cell subset associated with IgG/IgM production, could be induced after the *in vitro* co-culture of activated CD4^+^ T cells with autologous B cells.^20^ This B cell subpopulation was detectable in the peripheral of SLE patients as well with high levels of phosphorylated Syk and Erk1/2.^19^ It was also shown that CD19^hi^ B cells upregulated Blimp1 (Figures S2A and S2B), IRF4 (Figures S2C and S2D) and CD138 (Figures S2E and S2F) when compared to CD19^lo^ B cells, which displayed the phenotypic properties of plasma cells. Therefore, we analyzed the induction of CD19^hi^ B cells after the co-cultures of B cells with either 24 h or 72 h-stimulated CD4^+^ T cells. It was apparent that more CD19^hi^ B cells were induced after 24 h-stimulated CD4^+^ T-B co-cultures (Figure 1B). Consistent with different patterns of IgG production in CD4^+^ T-B cell co-cultures, there exhibited increased ratios of CD19^hi^/CD19^lo^ B cells in 24 h-stimulated CD4^+^ T-B co-culture than resting T cell-engaged co-culture whereas dramatic decrease in the ratios of CD19^hi^/CD19^lo^ B cells were observed in 72 h-stimulated CD4^+^ T-B co-culture (Figure 1C). Furthermore, we also found that absolute counts of CD19^hi^ B cell were increased after co-cultured with 24 h-stimulated CD4^+^ T cells whereas decreased with 72 h-stimulated CD4^+^ T cells (Figure 1D). Collectively, these data reveal an unusual phenomenon that CD4^+^ T cell activation upon longer TCR/CD28 stimulation (72 h) attenuates B cell differentiation and IgG production when compared to short duration of stimulation (24 h).

### Profiles of co-stimulatory molecule expression and cytokine production in human CD4^+^ T cells upon anti-CD3/CD28 stimulation

**Figure 2:**
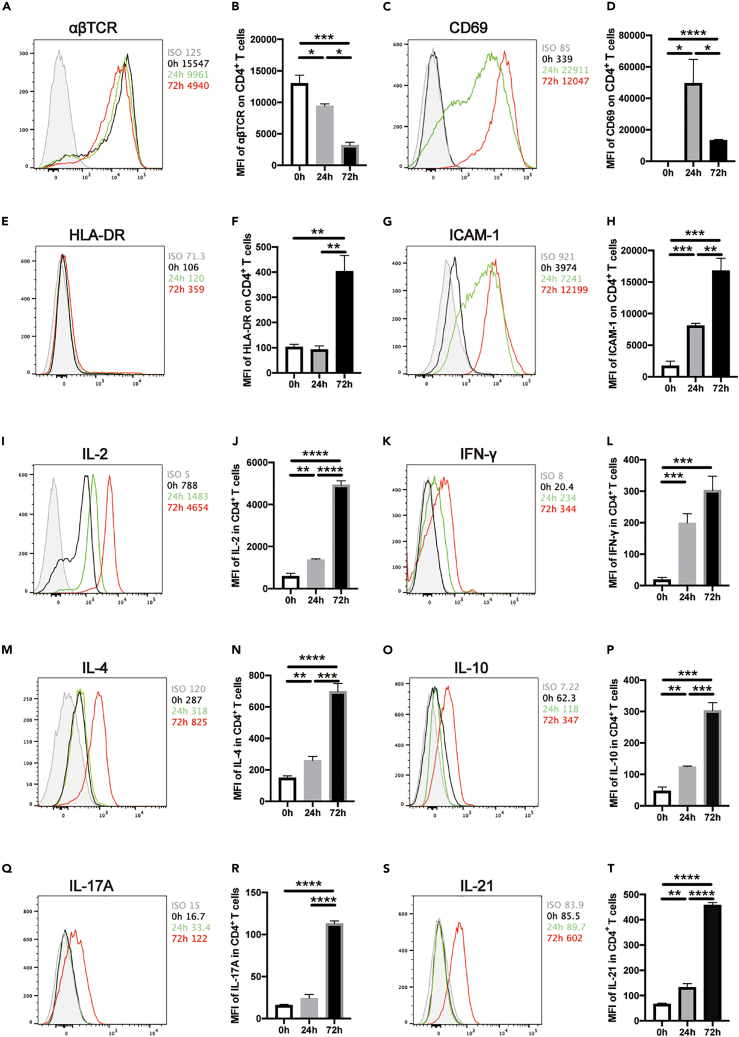
Profiles of co-stimulatory molecule expression and cytokine production in human CD4^+^ T cells upon anti-CD3/CD28 stimulation Purified human CD4^+^ T cells were stimulated with anti-CD3/CD28 beads for 24 h and 72 h, and subjected to phenotypic and functional analysis by flow cytometric analysis, including αβTCR (A-B), CD69 (C-D), HLA-DR (E-F), ICAM-1 (G-H), IL-2 (I-J), IFN-γ (K-L), IL-4 (M-N), IL-10 (O-P), IL-17A (Q-R) and IL-21 (S-T) (n = 6). The data were representative of at least three independent experiments. Data were represented by mean ± SD. The Kruskal-Wallis test with subsequent Duuns multiple-comparison test was used. *ns*: no significant; ∗: p < 0.05; ∗∗: p < 0.01; ∗∗∗: p < 0.001; ∗∗∗∗: p < 0.0001.

To explore the underlying mechanisms that longer activation of human CD4^+^ T cells dampens functional differentiation of B cells, we firstly examined the profiles of co-stimulatory molecules and cytokine production of CD4^+^ T cells upon anti-CD3/CD28 stimulation for 24 h and 72 h, respectively. It was obvious that upon TCR/CD3 stimulation, αβTCR levels on CD4^+^ T cells were dramatically decreased upon 24 h’ and 72 h’ stimulation (Figures 2A and 2B) due to the internalization.^20^ CD69 expression on CD4^+^ T cells, an early activation marker, increased dramatically after 24 h and decreased later on at 72 h (Figures 2C and 2D). HLA-DR expression, a late activation indicator, was upregulated along with stimulation duration (Figures 2E and 2F). We also found that ICAM-1 expressions on CD4^+^ T cells increased gradually with the elongation of stimulation duration (Figures 2G and 2H). Simultaneously, we detected key cytokine production in CD4^+^ T cells including IL-2 (Figures 2I and 2J), IFN-γ (Figures 2K and 2L), IL-4 (Figures 2M and 2N), IL-10 (Figures 2O and 2P), IL-17A (Figures 2Q and 2R) and IL-21 (Figures 2S and 2T). Most of cytokine production at 72 h was higher than that at 24 h upon anti-CD3/CD28 stimulation except IL-2. These results apparently indicate that upon TCR/CD28 stimulation *in vitro*, CD4^+^ T cells exhibit more activation status at 72 h than at 24 h with hyperactivation phenotypes.

### ICAM-1 is engaged in increased human CD4^+^ T cell-B cells adhesion upon TCR/CD28 stimulation

**Figure 3:**
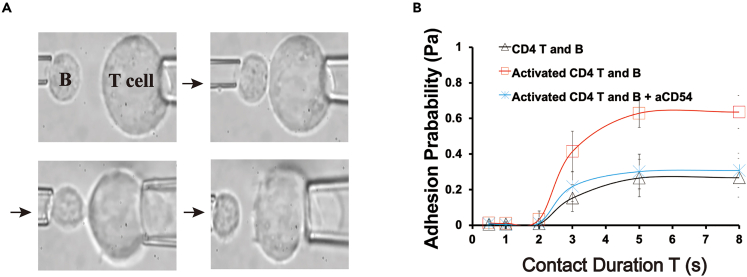
ICAM-1 is engaged in increased human CD4^+^ T cell-B cells adhesion upon TCR/CD28 stimulation (A) Scheme of micropipette adhesion frequency assay *in vitro*. (B) Adhesions between B cells and resting or activated CD4^+^ T cells from HCs with or without anti-ICAM-1 blocking mAb (5 μg/mL) by the micropipette adhesion frequency assay. The data were representative of at least three independent experiments. Data were represented by mean ± SD. The Kruskal-Wallis test with subsequent Duuns multiple-comparison test was used. ∗: p < 0.05; ∗∗: p < 0.01; ∗∗∗: p < 0.001.

T-B cell interaction is essential for T cell-dependent antibody responses. Investigations from mouse models illustrate that CD4^+^ T-B conjugates occur at the border area of the follicles (B cell zone) firstly and move to T cell zones after T cells are primed by the antigens.^21^^,^^22^^,^^23^ Our previous study indicated that during human CD4^+^ T-B cell interaction, ICOS and CD40L exerted distinct roles in cell-cell adhesion and B cell differentiation.^19^ ICAM-1 is one of key molecules to mediate cell-cell adhesion which should be engaged in the first step of T-B interaction. We therefore investigated how ICAM-1 regulated CD4^+^ T cell-B cell adhesion. To address this question, micropipette adhesion frequency assay (Figure 3A)^19^^,^^24^ has been adapted. Consistent with our previous study, when CD4^+^ T cells were activated with anti-CD3/CD28 beads, the adhesion frequencies (*Pa* values) (Figure 3B, red line) were significantly higher than those between resting CD4^+^ T cells and B cells (both p < 0.001) from the contacting time at 3 s (Figure 3B, blue line). When pre-incubating anti-ICAM-1 blocking mAb with activated CD4^+^ T cells, the *Pa* values decreased dramatically that was close to those of resting CD4^+^ T cells. When we performed flow cytometry to determine the molecular density of ICAM-1 on activated CD4^+^ T cells at different time point (Figure S1), it was indicated that ICAM-1 expression was upregulated on activated CD4^+^ T cells especially after 72 h stimulation (Figure S1D). These results thus indicate that there exhibits increased adhesion between B cells and CD4^+^ T cells once CD4^+^ T cells are activated upon TCR/CD28 signaling in which ICAM-1 is engaged in increased human CD4^+^ T-B cell adhesion.

### ICAM-1 blockade leads to increased IgG production by B cells when co-culturing with 72 h-stimulated CD4^+^ T cells

**Figure 4:**
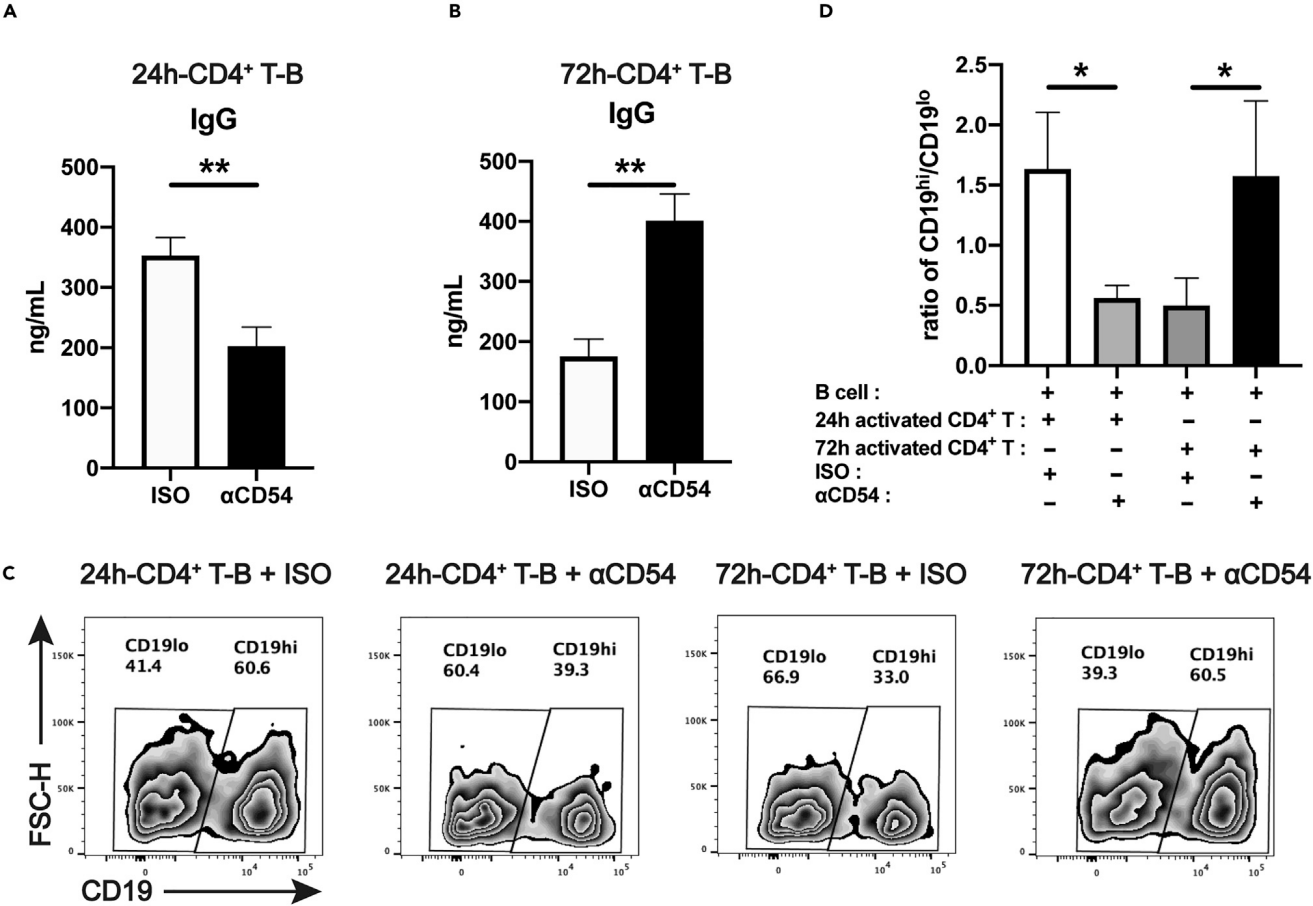
ICAM-1 blockade leads to increased IgG production when B cells are co-cultured with 72 hrs-stimulated CD4^+^ T cells (A and B) IgG levels in the supernatants of the co-cultures of B cells with 24 hrs- (A) or 72 hrs- (B) stimulated CD4^+^ T cells with isotype Ab or anti-ICAM-1 blocking mAb. (C) CD19^lo^ and CD19^hi^ B cell subpopulations after the co-culture of 24 hrs- or 72 hrs-stimulated CD4^+^ T cells at Day 12. (D) Comparisons on the ratios of CD19^hi^/CD19^lo^ B cells after the co-culture. The data were representative of at least three independent experiments. Data were represented by mean ± SD. In Figures 4A and 4B the paired *Student’s* t test was used. In Figure 4D, the Kruskal-Wallis test with subsequent Duuns multiple-comparison test was used. ∗: p < 0.05; ∗∗: p < 0.01.

Since cell-cell adhesion is mostly the first step for T cell-dependent B cell differentiation, we further investigated whether ICAM-1 played certain roles in IgG production through adding anti-ICAM-1 blocking mAb during CD4^+^ T-B cocultures. Human CD4^+^ T cells stimulated with anti-CD3/CD28 beads either for 24 h or 72 h were co-cultured with autologous B cells with or without anti-ICAM-1 mAb. IgG levels in the supernatants were determined after 12 days. It was found that IgG levels were decreased in the supernatants of 24 h-stimulated CD4^+^ T-B cell cultures with the blockade of ICAM-1 (Figure 4A) when compared to isotype controls, which suggests that ICAM-1-mediated adhesion facilitates IgG production. Surprisingly, addition of anti-ICAM-1 blocking mAb during the coculture led to the increase in IgG production after 12 days (Figure 4B), which is contrary to the results from CD4^+^T cells with the activation time for 24 h. The blockade of cell-cell adhesion with 72 h-stimulated CD4^+^ T cells was inclined to facilitate IgG production.

We also analyzed the induction of CD19^hi^ B cells after the co-cultures of 24 h or 72 h-stimulated CD4^+^ T cells and B cells with or without the blockade of ICAM-1. It was found that addition of anti-ICAM-1 blocking mAb in the 24 h-stimulated CD4^+^ T-B cell co-culture led to the dramatic decrease in CD19^hi^/CD19^lo^ B cell ratios after 12 days. However, CD19^hi^/CD19^lo^ B cell ratios after 72 h-stimulated CD4^+^ T-B cell co-culture increased significantly when compared to isotype controls (Figures 4C and 4D). These results were extremely consistent with IgG production we detected in the supernatants of CD4^+^ T-B co-cultures with two types of activated CD4^+^ T cells. Our data therefore strongly implies that ICAM-1 on activated CD4^+^ T cells might exert diverse roles in B cell differentiation and IgG production depending on activation times.

### Increased IgG production upon ICAM-1 blockade in 72 h-stimulated CD4^+^ T-B co-culture is Erk1/2 dependent

**Figure 5:**
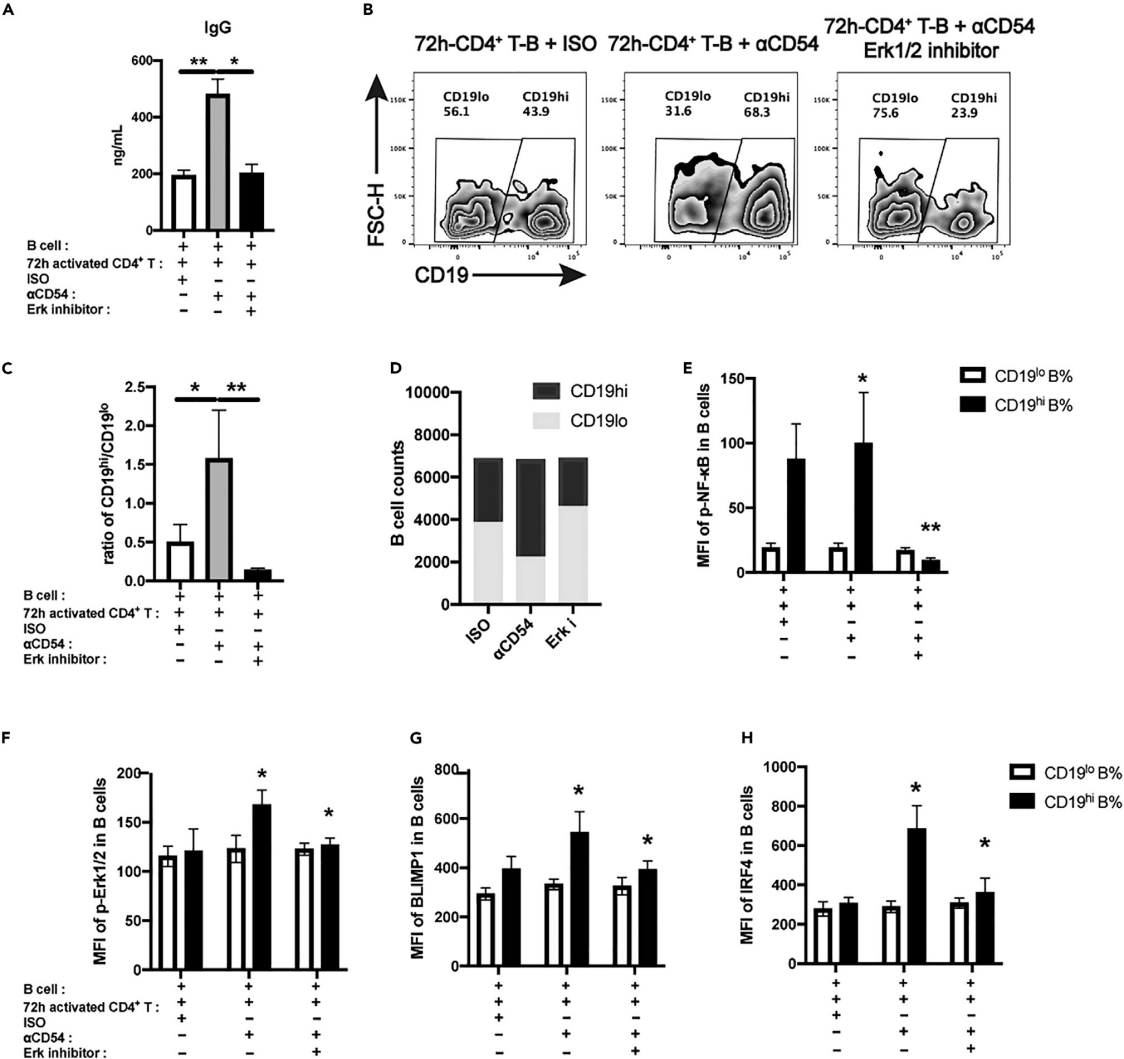
Increased IgG production upon ICAM-1 blockade in 72 hrs-stimulated CD4^+^ T-B co-culture is Erk1/2 dependent (A) IgG content in the supernatants of 72 hrs-stimulated CD4^+^ T-B cell co-culture upon ICAM-1 blockade with or without an Erk1/2 inhibitor. (B) Detection of CD19^lo^ and CD19^hi^ B cell subsets in the co-cultures at Day 12. (C) Comparisons on the ratios of CD19^hi^/CD19^lo^ B cells after the co-culture. (D) Absolute counts of CD19^lo^ and CD19^hi^ B cells after the co-culture. (E–H) The mean fluorescence intensity of phosphorylation of p-NF-κB p65 (pS529) (E), *p*-Erk1/2 (pT202/pY204) (F), Blimp1 (G) and IRF4 (H) in CD19^lo^ and CD19^hi^ B cell subsets after the co-culture for 12 days. The data were representative of at least three independent experiments. Data were represented by mean ± SD. The Kruskal-Wallis test with subsequent Duuns multiple-comparison test was used. ∗: p < 0.05; ∗∗: p < 0.01.

It is out-of-expectation that although CD4^+^ T cells upon 72 h activation displays more activation features including higher ICAM-1 expression, interruption of ICAM-1-mediated adhesion seems to promote B cell differentiation and IgG production. To validate this phenomenon from the viewpoint of intracellular signals, we first determined whether Erk1/2 activation was involved in ICAM-1 blockade-mediated IgG over-production in 72 h-stimulated CD4^+^ T-B co-culture. Erk1/2 activation is reported to be involved in B cell activation and proliferation.^25^^,^^26^ When an Erk1/2 inhibitor SCH772984 was added in 72 h-stimulated CD4^+^ T-B co-culture at a final concentration of 2 μM in the presence of anti-ICAM-1 blocking mAb, it was found that addition of SCH772984 dramatically diminished the increment of IgG production (Figure 5A) as well as the ratios of CD19^hi^/CD19^lo^ B cells when compared to anti-ICAM-1 mAb alone group (Figures 5B and 5C). Addition of SCH772984 slightly reduced the absolute number of CD19^hi^ B cells after the coculture (Figure 5D). Meanwhile, Erk1/2 inhibitor also dramatically suppressed the phosphorylation of NF-κB (p65) (Figure 5E) and Erk1/2 (pT202/pY204) (Figure 5F) in CD19^hi^ B cell subset. Both of them are reported to be involved in B cell activation and differentiation.^27^^,^^28^ However, phosphorylation of p65 was not altered in CD19^lo^ B cells. Blimp1 and IRF4 are key transcriptional factors to promote the differentiation of plasma cells.^29^^,^^30^ Erk1/2 inhibition also led to dramatic decrease in the expressions of Blimp1 and IRF4 in CD19^hi^ B cell subpopulation after the co-culture (Figures 5G and 5H). Taken together, our data demonstrate that ICAM-1 blockade-induced IgG hyper-production is Erk1/2 dependent, which is accompanied by increased phosphorylation of NF-κB and over-expression of Blimp1 and IRF4 in CD19^hi^ B cell subsets.

### PD-1/PD-L1 ligation attenuates ICAM-1 blockade-induced IgG over-production in 72 h-stimulated CD4^+^ T-B co-culture

**Figure 6:**
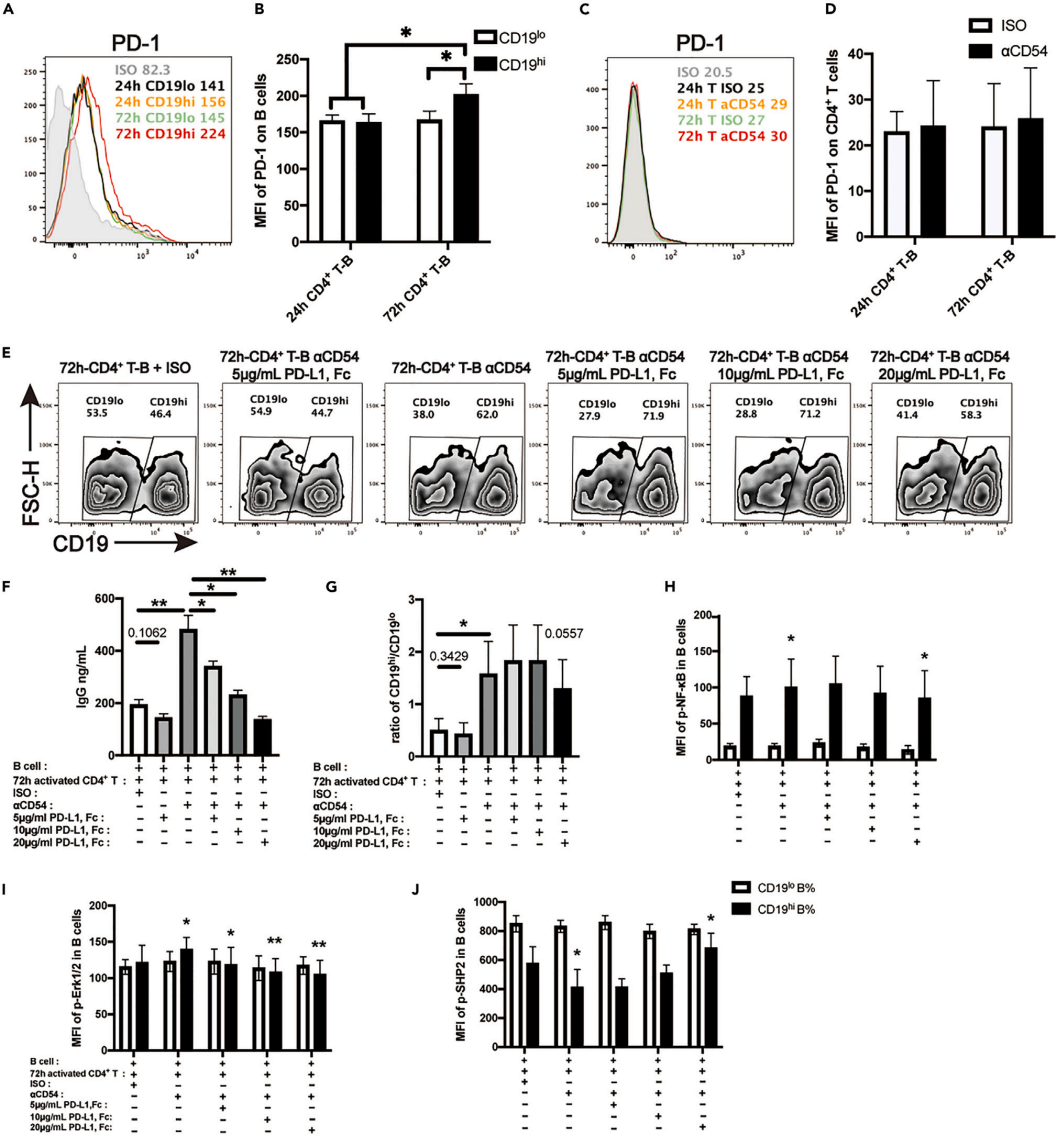
PD-1/PD-L1 ligation attenuates ICAM-1 blockade-related IgG hyper-production in 72 hrs-stimulated CD4^+^ T-B co-culture (A) PD-1 expressions on CD19^lo^ and CD19^hi^ B cells after 24 hrs- or 72 hrs-stimulated CD4^+^ T-B co-cultures for 12 days. (B) Comparisons on the percentages of PD-1^+^CD19^lo^ and PD-1^+^CD19^hi^ B cells after the co-cultures. (C) PD-1 expression on CD4^+^ T cells after 24 hrs- or 72 hrs-stimulated CD4^+^ T-B co-cultures for 12 days. (D) Comparisons on PD-1^+^CD4^+^ T cell percentages after co-cultures. (E) PD-L1 expression on CD4^+^ T cells after 24 hrs- or 72 hrs-stimulated CD4^+^ T-B co-cultures for 12 days. (F) IgG production in the supernatants of 72 hrs-stimulated CD4^+^ T-B cell co-culture upon ICAM-1 blockade with the addition of PD-L1-Fc proteins at different concentrations (5, 10, 20 μg/mL). (G) The percentages of CD19^lo^ and CD19^hi^ B cell subpopulations in the co-cultures at Day 12. (H–J) The MFI of phospho-NF-κB p65 (pS529) (H), phospho-Erk1/2 (pT202/pY204) (I) and phospho-SHP-2 (Y542) (J) levels in CD19^lo^ and CD19^hi^ B cell subsets after co-culturing for 12 days. The data were representative of at least three independent experiments. Data were represented by mean ± SD. The Kruskal-Wallis test with subsequent Duuns multiple-comparison test was used. ∗: p < 0.05; ∗∗: p < 0.01.

Since blockade of ICAM-1 might interrupt CD4^+^ T-B cell adhesion (Figure 3), we speculated that interruption of CD4^+^ T-B adhesion might also affect certain signals negatively regulating B cell differentiation and IgG production. We therefore determined the expression of PD-1 on B cells after co-culturing with either 24 h or 72 h-stimulated CD4^+^ T cells. It was shown that while no difference in PD-1 expression on CD19^lo^ B cells was observed after the co-culture with 24 h or 72 hrs-stimulated CD4^+^ T cells, PD-1 expression on CD19^hi^ B cells was increased dramatically when co-culturing with 72 h-stimulated CD4^+^ T cells compared to those with 24 h-stimulated CD4^+^ T cells (Figures 6A and 6B). However, PD-1 expression on CD4^+^ T cells was comparable after the co-cultures (Figures 6C and 6D). To validate whether PD-1/PD-L1 ligation is involved in regulating IgG production upon ICAM-1 blockade, we added PD-L1-Fc proteins at the concentrations of 5, 10 and 20 μg/mL respectively in 72 h-stimulated CD4^+^ T-B co-cultures with anti-ICAM-1 blocking mAb. After 12 days, it was found that addition of PD-L1-Fc protein dramatically reduced IgG production in the co-culture supernatants in a dose dependent manner (Figure 6F). However, it did not significantly affect the ratios of CD19^hi^/CD19^lo^ B cells after the co-culture in the presence of anti-ICAM-1 blocking mAb (Figures 6E and 6G). Noteworthy, PD-L1-Fc protein alone only had slightly decreased IgG production (Figure 6F) with no effects on the ratios of CD19^hi^/CD19^lo^ B cells (Figures 6E and 6G).

Consistently, addition of PD-L1-Fc protein in the ICAM-1 blockade co-culture led to the decrease in the phosphorylation of NF-κB (p65) (Figure 6H) and Erk1/2 (pT202/pY204) (Figure 6I) in CD19^hi^ B cell subpopulation after the co-culture. We also detected phosphorylation levels of SHP-2, a protein phosphatase to dephosphorylate NF-κB (p65) and Erk1/2 (pT202/pY204),^31^^,^^32^ in CD19^hi^ B cell subpopulation. It was found that phospho-SHP-2 (Y542) levels were increased in CD19^hi^ B cell subpopulation with the addition of PD-L1-Fc protein in a dose dependent manner (Figure 6J). Collectively, our data support that PD-1 expression is up-regulated on activated B cells after coculturing with 72 h-stimulated CD4^+^ T cells with more extent. ICAM-1 blockade likely interrupts PD-1-PD-L1 ligation, which in turn attenuates negative signaling for B cell differentiation and IgG production and leads to the increased IgG production we observed in 72 h-stimulated CD4^+^ T-B co-culture upon ICAM-1 blockade.

### High expression of ICAM-1 on CD4^+^ T cells from moderate/severe SLE patients also mediates suppression on IgG production after T-B coculture

**Figure 7:**
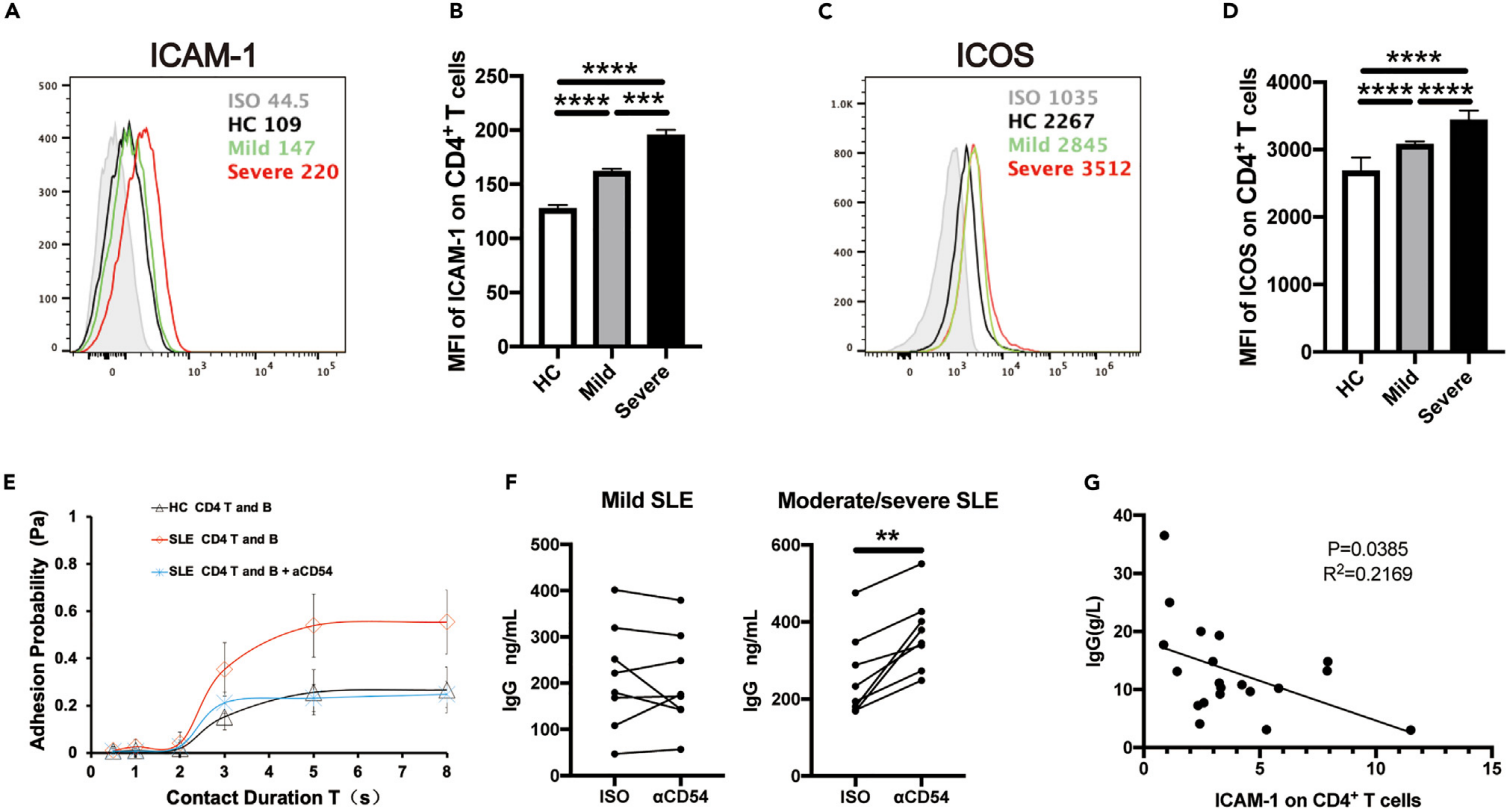
High expression of ICAM-1 on CD4^+^ T cells from moderate/severe SLE patients mediates the suppression on IgG production after T-B coculture (A–D) Flow cytometric analysis of the percentages of ICAM-1 (A and B) and ICOS (C and D) expressing CD4^+^ T cells in mild and moderate/severe SLE patients or HCs. (E) Adhesions between autologous B cells and pathogenic CD4^+^ T cells from SLE patients with anti-ICAM-1 blocking mAb (5 μg/mL). (F) IgG contents in the supernatants of pathological T-B co-cultures upon ICAM-1 blockade. (G) Correlation between IgG contents and the percentages of ICAM-1^+^CD4^+^ T cells from SLE patients. The data were representative of at least three independent experiments. Data were represented as mean ± SD. In Figures 7B and 7D, the Kruskal-Wallis test with subsequent Duuns multiple-comparison test was used. In Figure 7F, the Wilcoxon matched paired test was used. In Figure 7G, the Pearson correlation analysis was used. ∗∗: p < 0.01; ∗∗∗: p < 0.001; ∗∗∗∗: p < 0.0001.

SLE is an autoimmune disease characterized by autoantibody-driven tissue and organ damages. Emerging evidence reveals that exaggerated B cell immune responses play central roles in the pathogenesis of SLE. In fact, we have also observed dramatic increase in ICAM-1 (Figures 7A and 7B) and ICOS (Figures 7C and 7D) expressions on peripheral CD4^+^ T cells from moderate/severe SLE patients when compared to HCs and mild SLE, which indicated the hyper-activation of CD4^+^ T cells in moderate/severe SLE.

By using the micropipette adhesion frequency assay, we have observed increased adhesion between freshly isolated CD4^+^ T cells and B cells from SLE patients (Figure 7E, red line) when compared to that from HC counterparts (Figure 7E, black line). When pre-incubating CD4^+^ T cells with anti-ICAM-1 blocking mAb, there exhibited the decreased *Pa* values in the CD4^+^ T cell-B cell adhesion assay (Figure 7E, blue line), demonstrating the involvement of ICAM-1 in increased cell-cell adhesion under SLE pathogenesis.

We further performed ICAM-1 blockade in CD4^+^ T-B co-cultures from either mild or moderate/severe SLE patients whose ICAM-1 expression levels were different. Consistent with the results from healthy donors, blockade of ICAM-1 in CD4^+^ T-B cell coculture from moderate/severe SLE patients also led to the increase in IgG production (Figure 7F, right) whereas no difference existed in those from mild SLE patients with lower expression of ICAM-1 on CD4^+^ T cells (Figure 7F, left). These results to some extent recapitulated the phenomenon we observed in healthy donors. Furthermore, we also found that the percentages of peripheral ICAM-1^+^CD4^+^ T cells had significantly negative correlations with IgG contents in SLE patients (Figure 7G). The data from SLE patients further support that high expression of ICAM-1 on over-activated CD4^+^ T cells promotes T-B cell adhesion whereas mediates negative signals for B cell differentiation and IgG production.

## Discussion

T-B cell interactions are critical for T cell-dependent B cell activation and terminal differentiation as well as IgG generation mostly through the ligations of co-stimulatory molecules.^33^ ICAM-1 is a key adhesion molecule to mediate cell-cell adhesion. Previous studies have revealed that ICAM-1 was engaged in long-lasting cognate T-B interactions necessary for the selection of low-affinity B cell clones in T cell-dependent antibody responses.^10^ In this study, we have reported an extraordinary phenomenon that ICAM-1 expression on hyper-activated CD4^+^ T cells (anti-CD3/CD28 activation for 72 h) mediated strong cell-cell adhesion whereas unexpectedly dampened B cell differentiation and IgG production. This is largely due to inducible expression of PD-1 on CD19^hi^ B cells after T-B co-culture mediating negative signals to suppress IgG production. We therefore intend to propose dual modules of ICAM-1-engaged adhesion between activated CD4^+^ T cells and B cells for B cells differentiation and IgG production probably relying on inducible expression of certain negative regulatory molecules such as PD-1 on B cells. Since PD-1 expression is induced along with CD4^+^ T-B interactions, our results imply a novel intrinsic mechanism to regulate B cell differentiation and IgG production through cell-cell adhesion.

We observed this phenomenon unexpectedly in the longitude investigations to decipher the effects of cell-cell adhesion on B cell differentiation. In fact previous investigations revealed the critical roles of cell-cell adhesion in promoting T cell activation,^34^ cytotoxicity of NK cells^35^ as well as B cell differentiation in GC.^30^ Cell-cell adhesion occurs at the early stage of immune responses. Formation of immune cell conjugates not only establishes direct liaisons between targeted immune cells, but also delivers key signals to determine whether immune cells undergo activation or not through the ligations between multiple molecule pairs.^36^ In most cases immune responses will not be triggered without direct cell-cell contact. Being one of the key adhesion molecules, ICAM-1 is demonstrated to be engaged in cell-cell adhesion mainly.^37^ Herein, we have observed dramatic upregulation of ICAM-1 on human CD4^+^ T cells once activated by anti-CD3/CD28 *in vitro* with more adhesion frequencies between CD4^+^ T cells and B cells (Figure 3). However, increased adhesions between activated CD4^+^ T cells and B cells are not aligned with increased IgG production in our study (Figure 1A). Longer activated duration CD4^+^ T cells undergo, less IgG B cells produce. We therefore deduced that cell-cell adhesion of B cells with hyper-activated CD4^+^ T cells might delivery certain negative signals to restrain B cell activation and differentiation.

We have further demonstrated that inducible expression of PD-1 on B cells after encountering activated CD4^+^ T cells was one of the mechanisms to regulate human B cell differentiation and IgG production negatively. PD-1 is inducibly expressed on T cells and exerts co-inhibitory effects to restrain T cell immunity especially in anti-tumor immunity.^38^^,^^39^ It can recruit protein diphosphatase SHP-2 to dephosphotate activated signal molecules downstream TCR signal including CD3, ZAP-70 et al. to suppress T cell activation.^40^ Several studies have reported the roles of PD-1 in B cell activation and differentiation in mouse models. For instance, through inducing more cell death in GCs and less cytokine production by Tfh cells, PD-1 is dedicated to optimal formation of long-lived plasma cells by regulating the survival and selection of B cells within GCs.^41^ PD-1 is also required for optimal IL-21 production by Tfh cells.^41^^,^^42^ We have previous reported that injection of PD-1 blocking antibodies in OVA immunized mice led to the augmentation of total IgG and OVA-specific antibodies,^43^ supporting negative regulation of PD-1 on B cell differentiation. It is also reported that depletion of PD-1 promoted Tfh cell expansion in vaccine-immunized mice via promoting cytokines secretion and enhanced humoral immunity to Malaria infection.^44^^,^^45^ In mechanism exploration, Shi et al. reported that PD-1 suppressed the recruitment of Tfh cells but promoted the numbers of Tfh cells in the GC territory, which helped to maintain the stringency of GC affinity selection.^42^ These data have outlined the direct roles of PD-1 in regulating B cell differentiation and IgG production in mouse GCs. In our study, based on the *in vitro* human T-B coculture assay, we have observed the up-regulation of PD-1 expression on CD19^hi^ B cells, a subpopulation responsible for IgG production,^19^^,^^20^ when coculturing with 72 h-stimulated CD4^+^ T cells. The upregulation of PD-1 on activated B cells could interact with PD-L1 to transmit a negative signal and attenuate B cell differentiation and IgG production where ICAM-1 was largely responsible for cell-cell contact to bridge PD-1 and PD-L1 ligation.

The results from molecular mechanism exploration are also consistent with the observations on B cell differentiation and IgG production upon ICAM-1 blockade. Collectively, Erk1/2 and NF-κB activation were detectable upon ICAM-1 blockade together with Blimp1 and IRF4 upregulation in CD19^hi^ B cell subset in 72 h-stimulated CD4^+^ T-B cell coculture. They are key contributors to B cell differentiation and antibody production.^46^^,^^47^ When PD-L1-Fc fusion protein was added in the blocking assays, the activation status was apparently reversed whereas SHP-2 phosphorylation was up-regulated. Since no dramatic difference of PD-1 expression on CD4^+^ T cells is observed after the co-culture with or without ICAM-1 blockade (Figures 6C and 6D), the inducible expression of PD-1 on CD19^hi^ B cells is probably an intrinsic key event after B cells contact with activated CD4^+^ T cells to regulate B cell activation and differentiation. When cell-cell adhesion is interrupted by ICAM-1 blockade, this negative feedback is impaired which leads to over-activation of B cells and subsequent IgG hyper-production that we observed in 72 h-stimulated CD4^+^ T-B cell coculture.

It is well-known that ICAM-1 is also expressed on B cells. However, the expression patterns of ICAM-1 on B cells and T cells are different according to our results. B cells express ICAM-1 constitutively. Even after 12 days of T-B co-culture, the expression levels of ICAM-1 on B cells are comparable (Figure S2G). However, we have observed dramatic upregulation of ICAM-1 on CD4^+^ T cells upon anti-CD3/CD28 stimulation, which could represent the activation status as well as functional implementation of ICAM-1 for CD4^+^ T cell activation to some extent. Therefore, the effects of ICAM-1 blockade might be performed on both B cells and CD4^+^ T cells where it is more dramatic on CD4^+^ T cells. We herein propose that up-regulated ICAM-1 on CD4^+^ T cells should serve as a bridge mediating T-B adhesion, which is critical for both co-stimulatory and co-inhibitory molecules to deliver diverse signals for B cell differentiation in our system. What is more, in our *in vitro* co-culture system B cell activation and differentiation are non-antigen specific. We therefore would like to propose that current CD4^+^ T-B co-culture model is similar to T-B interactions in extrafollicular GC responses in mouse models with less antigen-dependence.^48^^,^^49^ It might represent the situation at the early stage of T cell-dependent B cell activation when activated CD4^+^ T cells initiate B cells differentiation. Although BCR signal is not involved, increased adhesion through ICAM-1 could be more feasible for cytokine and co-stimulatory or co-inhibitory signaling to regulate B cell differentiation and IgG production. The direct relationship between ICAM-1-mediated ligations and differentiation phenotypes of B cells needs to be further investigation.

Since either membrane or soluble ICAM-1 is prominent in SLE patients from our investigations and others,^50^ we also explored whether activated CD4^+^ T cells from SLE patients functioned similarly to affect B cell differentiation and IgG production. CD4^+^ T cells from moderate-to-severe SLE patients with high ICAM-1 expression triggered comparable IgG production to those from mild SLE patients. However, blockade of ICAM-1 with the decrease in cell-cell adhesion promoted IgG production from moderate-to-severe SLE patients, which could be mirrored with the results from 72 h-stimulated CD4^+^ T cells from healthy donors. The significance might lie in the fact that although auto-reactive CD4^+^ T cells are hyper-activated under pathogenic status to promote B cell activation and auto-antibody production, they still possess intrinsic feedback mechanisms to control autoimmune responses to a less degree. The results we obtained may provide new clues for clinic to exaggerate PD-1 mediated inhibition as a new therapeutic strategy for the suppression of B cell over-activation in SLE.

Taken together, by using a well-established *in vitro* T-B co-culture system, we have illustrated that ICAM-1-mediated cell-cell adhesion could exert either positive or negative regulations during human CD4^+^ T cell and B cell interactions. Inducible expression of PD-1 on CD19^hi^ B cells accounts for negative feedback during B cell differentiation and IgG production after co-culturing with hyper-activated CD4^+^ T cells (Figure 6A). Based on our results, we would deduce that moderate activation of CD4^+^ T cells by the antigens is prone to introduce optimal B cell activation and differentiation whereas over-activation of CD4^+^ T cells will retard B cell activation. This might be helpful in vaccine designing to screen antigenic proteins or peptides with moderate immunogenicity for optimal humoral immunity. Among this process, ICAM-1 mediated T-B adhesion probably becomes a unique mechanism to regulate CD4^+^ T cell-guided B cell differentiation under given situations. The exact mechanisms of how activation status of CD4^+^ T cell regulating PD-1 expression on B cells needs to be investigated in the future.

### Signifinance

CD4^+^ T cell-B cell adhesion facilitates signal transduction to promote B cell activation and differentiation. Herein we have reported that although activated human CD4^+^ T cells upregulated ICAM-1 expression to promote cell-cell adhesion with autologous B cells, ICAM-1-engaged cell-cell adhesion also acted as one of attenuating mechanisms for B cell differentiation and IgG production partially through inducible expression of PD-1 on B cells and downstream negative signal transduction once CD4^+^ T cells were over-activated. Therefore, proper activation of CD4^+^ T cells would be beneficial for optimal B-cell terminal differentiation and IgG secretion, which might be helpful for long-term efficacy of humoral immunity.

### Limitations of the study

Our findings have revealed the engagement of ICAM-1 in CD4^+^ T-B adhesion in negative signal transduction during B-cell differentiation and IgG secretion. Nevertheless, our study has several limitations. We did not clarified that ICAM-1 on CD4^+^ T cells or B cells exerts more dominant roles to mediate human T-B adhesion and subsequent regulation, which could use CRISPR-Cas9 system to knock out the expressions of *ICAM-1* on human CD4^+^ T cell or B cell seperately. Othewise, we can also validate the *in vivo* regulatory modes of ICAM-1 on T-B adhesion and B cell differentiation in GCs via using CD4^Cre^ICAM-1^f^^^l^^^/f^^l^ mice. Moreover, we can not exclude the possibility that other negative co-inhibitory molecules may cooperate (or even synergize) with PD-1 signal to regulate IgG production, warranting additional mechanistic investigation. Finally, we could provide more evidence to address whether PD-1 agonist would inhibit pathological B cell differentiation and autoantibody production under SLE pathogenesis in the future.

## STAR★Methods

### Key resources table

**Table:** 

REAGENT or RESOURCE	SOURCE	IDENTIFIER
**Antibodies**		

Anti-human-CD3-PerCP/Cy5.5	BioLegend	Cat# 300328, RRID: AB_1575008
Anti-human-CD4-BV711	BD Biosciences	Cat# 563913, RRID: AB_2738484
Anti-human-CD4-BV510	BD Biosciences	Cat# 562970, RRID: AB_2744424
Anti-human- CD19-BV605	BD Biosciences	Cat# 562653, RRID: AB_2722592
Anti-human-CD69-PE	BioLegend	Cat# 310906, RRID: AB_314841
Anti-human-CD54-FITC	BioLegend	Cat# 353108, RRID: AB_10900254
Anti-human-CD54-PE	BioLegend	Cat# 353106, RRID: AB_10897647
Anti-human-αβTCR-APC	BioLegend	Cat# 109212, RRID: AB_313435
Anti-human-HLA-DR-APC/Cy7	BD Biosciences	Cat# 561358, RRID: AB_10611876
Anti-human-PD-1-BV650	BioLegend	Cat# 329906, RRID: AB_940483
Anti-human-IL-2-AF700	BioLegend	Cat# 500320, RRID: AB_528929
Anti-human-IL-4-APC	BioLegend	Cat# 500811, RRID: AB_315130
Anti-human-IL-10-PE/Cy7	BioLegend	Cat# 501420, RRID: AB_2125385
Anti-human-IFN-γ-PE	BD Biosciences	Cat# 559327, RRID: AB_397224
Anti-human-IL-17A-BV786	BioLegend	Cat# 512338, RRID: AB_2566765
Anti-human-IL-21-AF647	BD Biosciences	Cat# 560493, RRID: AB_1645421
Anti-human-ERK1/2 (pT202/pT204)-BV421	BD Biosciences	Cat# 562981, RRID: AB_2737930
Anti-human-NF-κB-p65 (pS529)-PE/Cy7	BD Biosciences	Cat# 560335, RRID: AB_1645545
Anti-human-SHP2 (Y542)-PE	BD Biosciences	Cat# 560389, RRID: AB_1645530
Anti-human-Blimp1-AF647	BD Biosciences	Cat# 565002, RRID: AB_2739040
Anti-human-IRF4-PE	BioLegend	Cat# 646404, RRID: AB_2563005

**Chemicals, reagents and recombinant proteins**		

TRIzol Reagent	Invitrogen	Cat# 15596018
Trypan Blue	Gibco	Cat# 15250061
Recombinant Human PD-L1-Fc	SinoBiological	Cat# 10084-H02H
Fixation/Perm Diluent	eBioscience	Cat# 00-5223-56
Fixation/Permeabilization Concentrate	eBioscience	Cat# 00-5123-43
Permeabilization buffer 10X	eBioscience	Cat# 00-8333-56
Dynabeads™ Human T-Activator CD3/CD28	Gibco	Cat# 11131D
ERK1/2 inhibitor (SCH772984)	Selleck	Cat# S7101
DMSO	Sigma	Cat# D2650
RPMI 1640 Medium	Gibco	Cat# 11875119
Fetal Bovine Serum	Gibco	Cat# 10099141C
Penicillin-Streptomycin (10000U/mL)	Gibco	Cat# 15140122

**Critical commercial assays**		

Human Immunoglobulin G (IgG) ELISA Kit	Elabscience	Cat# E-EL-H0169c
Human Immunoglobulin M (IgM) ELISA Kit	Elabscience	Cat# E-EL-H1814c
EasySep™ Human Naïve CD4^+^ T Cell Isolation Kit II	STEMCELL	Cat# 17555
EasySep™ Human Naïve B Cell Isolation Kit	STEMCELL	Cat# 17254

**Software and algorithms**		

Prism GraphPad (Version 8.0)	GraphPad Prism software	https://www.graphpad.com/scientific-software/prism
FlowJo (Version 10.0)	Tree Star software	https://www.flowjo.com/
Photoshop (Version CS6)	Adobe Systems	https://www.adobe.com/products/photoshop.html

### Resource availability

#### Lead contact

Further information and requests for reagents and resources should be directed and will be fulfilled by the lead contact, Ying Wang (ywang@sibs.ac.cn).

#### Materials availability

All the stable reagents generated in this study are available from the lead contact with a completed Materials Transfer Agreement.

#### Data and code availability

The dataset involving SLE patients subjects’ information containing age, gender and autoantibody titers is summarized in Table S1. Data reported in this paper will be shared by the lead contact upon request. This paper also not report original code. Any additional information required to reanalyze the data reported in this paper is available from the lead contact upon request.

### Experimental model and subject details

#### Human subjects

Healthy controls (HCs) were blood donor volunteers from Shanghai Blood Center with no history of medical diseases. All SLE patients were in-patient patients from Ruijin Hospital affiliated to Shanghai Jiao Tong University School of Medicine. They fulfilled the American Rheumatism Association criteria for the diagnosis of SLE (Table S1). All SLE patients have signed the informed consent forms individually. The study has been approved by the Ethic Committee of Shanghai Jiao Tong University School of Medicine. All the experiments were performed by the protocols according to the principles of the Declaration of Helsinki. Additional clinical information about the subjects is listed in *SI Appendix,* Table S1.

### Method details

#### Purification of human CD4^+^ T cells and B cells

Human whole blood was collected in heparin-treated tubes and peripheral blood mononuclear cells (PBMCs) were isolated by the density gradient centrifugation using LymphoprepT^M^ reagent (Axis-shield, Oslo, Norway). CD4^+^ T cells and CD19^+^ B cells were isolated by magnetic microbeads according to the manufacturer’s instructions (EasySep™ Human CD4 Positive Selection Kit II and EasySep™ Human CD19 Positive Selection Kit II, STEMCELL Technologies, Vancouver, Canada). Briefly, human PBMCs were mixed with antibody cocktail conjugated with biotin followed by the incubation with anti-biotin microbeads. The labeled cell mixtures were incubated in a EasySep™ magnet for 3 min. The unbound cells were carefully pipetted from the supernatants and discarded. The tubes containing CD4^+^ T cells or CD19^+^ B cells were removed from the magnet and washed once with Magnetic Cell Sorting (MACS) buffer. The purity of CD4^+^ T cells and CD19^+^ B cells were detected by flow cytometry using anti-human CD4-Percp-Cy5.5 and anti-human CD19-BV605 antibodies (Abs) (both from BD Biosciences, San Diego, CA, USA), respectively.

#### Activation of human CD4^+^ T cells *in vitro*

Purified CD4^+^ T cells from HCs were stimulated with Dynbeads™ Human T-Activator CD3/CD28 (bead-to-cell ratio 1:1) (Life Technologies, Carlsbad, CA, USA) in RPMI 1640 medium containing 10% fetal bovine serum (FBS), 100 units/mL penicillin and 100 μg/mL streptomycin (all from Life Technologies) in 96-well U-bottom plates (Thermo Fisher Scientific, Waltham, MA, USA). After 24 hrs’ or 72 hrs’ stimulation, the Dynabeads were removed and the cells were subjected to phenotypic analysis, adhesion assay and T-B coculture *in vitro*.

#### CD4^+^ T-B cell co-culture *in vitro*

CD4^+^ T cells (2 × 10^4^) from HCs with or without anti-CD3/CD28 stimulation were co-cultured with autologous B cells (5 × 10^4^) in 200 μL RPMI 1640 complete medium for 12 days. For SLE samples, freshly purified B cells were co-cultured with autologous CD4^+^ T cells for 12 days. The culture supernatants were collected for IgG detection. The remaining cell mixtures were subjected to phenotypic and functional analysis.

In the blockade assay, function-grade mouse anti-human ICAM-1(eBioscience, San Diego, CA, USA) mAb was added at the concentration of 5 μg/mL in the co-culture system. Erk1/2 inhibitor (SCH772984) (Selleck Chemicals, Houston, TX, USA) was added at the final concentration of 2 μM. PD-L1-Fc protein (Sino Biological, Beijing, China) was added at the final concentrations of 5, 10 and 20 μg/mL in the co-culture.

#### Micropipette adhesion frequency assay

The adhesion between CD4^+^ T and B cells was also measured by using a micropipette adhesion frequency assay modified from the protocol described previously.^19^^,^^51^ Briefly, one B cell and one CD4^+^ T cell were aspirated by two apposite micropipettes with the diameters of 3-5 μm in an isotonic chamber containing RPIM 1640 medium plus 5% FBS. The adhesion between one CD4^+^ T cell and one B cell was detected by placing the cells into controlled contact via driving in and out of the contact with controlled area and duration. The presence of adhesion at the end of a given contact time was detected by observing the deflection of the membrane of B cell or T cell when CD4^+^ T cell was retracted. This approach-contact-retraction cycle was repeated 100 times for one pair of B cell and CD4^+^ T cell at a given contact time. For each subject at an indicated given contact time, at least 3-5 pairs of one CD4^+^ T cell and one B cell were tested to calculate an adhesion probability (*Pa*) represented by the ratio of adhesion events to total events. In blocking experiments, mouse anti-human ICAM-1 mAb (5 μg/mL) (eBioscience) were pre-incubated with CD4^+^ T cells at 37 °C for 30 min before the adhesion assay.

#### Detection of molecular density

Molecular density of ICAM-1 on human CD4^+^ T cells was determined by flow cytometry as described previously.^24^ In brief, CD4^+^ T cells were incubated with anti-human ICAM-1-PE Ab (BD Biosciences) in FACS buffer (PBS containing 1% FBS and 1 mM EDTA) at 4°C for 30 min. Cell mixtures were washed once with PBS and resuspended in 200 μL FACS buffer. Cells were acquired by BD LSR Fortessa flow cytometry (BD Biosciences) along with four standard calibration beads (Quantibrite PE beads, from BD Biosciences). A calibration curve of PE molecules/beads (provided by manufacturer) (Figure S1A) *vs* mean fluorescence intensity (MFI) of anti-human ICAM-1-PE (Figure S1B) was plotted based on the curve of Quantibrite PE beads (*filled circles*, Figure S1C). The density of ICAM-1 on human CD4^+^ T cells was calculated by comparing the MFI of the sample (*open square,* Figure S1C) with the calibration curve.

#### Flow cytometry

Cells were resuspended in 50 μL FACS buffer and incubated with fluorochrome-conjugated mAbs at 4°C for 30 min, including mouse anti-human CD3-Percp-Cy5.5, anti-human CD4-BV711, anti-human CD19-BV605, anti-human αβTCR-APC, anti-human CD69-PE (all from eBioscience), anti-human HLA-DR-APC-H7, anti-human CD3-Percp-Cy5.5, anti-human CD4-BV510, anti-human PD-1-BV650 (all from BD Biosciences) or anti-human ICAM-1-FITC (Biolegend, San Diego, CA, USA). Intracellular signaling molecule detection was performed by using the Transcription Factor Buffer Set (BD Biosciences) following the manufacturer’s instruction. Briefly, cells were fixed and permeabilized by TF Fix/Perm Solution in the dark for 45 min at 4°C, followed by washing with 1 × TF Perm/Wash Buffer. Cells were stained with anti-Erk1/2 (pT202/pY204)-BV421, anti-SHP2 (Y542)-PE, anti-NF-κB-p65 (pS529)-PE-Cy7 and anti-AID-AF647 (all from BD Biosciences) for 45 min at 4°C. After washing with TF Perm/Wash Buffer, cells were resuspended in 200 μL FACS buffer and acquired on BD LSR Fortessa™ (BD Biosciences). Data were analyzed by using the FlowJo 10.5.0 software (Tree Star Inc.).

For cytokine staining, CD4^+^ T cells were fixed and permeabilized by using Fix/Perm Solution for 20 min. Cells were washed once with Perm/Wash Buffer, and were incubated with mouse anti-human IL-2-AF700, anti-human IL-4-APC Abs (all from Biolegend), mouse anti-human IL-10-PE-Cy7 Ab (eBioscience), anti-human IFN-γ-PE, anti-human IL-17A-BV786 and anti-human IL-21-AF647 Abs (all from BD Biosciences) at 4°C for 45 min. After washing once with Perm/Wash Buffer, CD4^+^ T cells were resuspended in 200μL FACS buffer and acquired on BD LSR Fortessa™ (BD Biosciences) and data analysis was performed by using FlowJo 10.5.0 software (Tree Star Inc.)

#### Enzyme-linked immunosorbent assay (ELISA)

The co-culturing supernatants were subjected to IgG quantification according to the manufacture’s instruction (Sen Xiong Biotech., Shanghai, China). The absorbance at 450 nm was detected within 5 min by the Power Wave XS2 microplate spectrophotometer (BioTek, VT, USA). The concentrations of IgG in the supernatants were calculated based on the standard curves.

### Quantification and statistical analysis

All statistical analyses were performed with the GraphPad Prism 8.0 (GraphPad Software, Inc., San Diego, CA, USA). Graphs were created using the GraphPad Prism 8.0 and edited using Adobe Illustrator CS6 (Adobe Inc., San Jose, CA, USA). Data were presented as means ± standard deviation (S.D). The normality of the data was evaluated by the Shapiro-Wilk normality test. If the data did not follow the normal distribution or the normality test indicated that the number was too small, the Mann-Whitney U or Wilcoxon matched paired test, or the Kruskal-Wallis test with subsequent Duuns multiple-comparison test was used to calculate the statistics difference. Otherwise, the unpaired or paired *Student’s t*-test, or one-way ANOVA with subsequent Tukey’s post-tests, was performed. Unless stated, *p* < 0.05 was considered statistically significant.
