# Supplementary material for: ICAM-1 mediated cell-cell adhesion exerts dual roles on human B cell differentiation and IgG production
Source: iScience. 2023 Nov 22;26(12):108505. doi: 10.1016/j.isci.2023.108505 (PMC10755720; doi:10.1016/j.isci.2023.108505)
Supplement: Document S1. Figures S1–S3 and Table S1 [file mmc1.pdf]

iScience, Volume *26*

Supplemental information

ICAM-1 mediated cell-cell adhesion exerts dual roles on human B cell differentiation and IgG production

Shuai Liu, Zhi-cui Liu, Mei-yu Zhang, Shu-jun Wang, Meng Pan, Ping Ji, Cheng Zhu, Ping Lin, and Ying Wang

Supplemental Information

Table S1. Clinical information of 38 SLE patients involved in the study, Related to Figure 7

Number	Gender	Age	C3 (79-152 mg/dl)	C4 (16-38 mg/dl)	IgG (751-1560mg/dl)	IgM (46-304 mg/dl)	IgA (82-453 mg/dl)	ANA	anti-dsDNA (< 100 IU/ml)
1	F	57	72	21	1540	80	432	1: 320	638
2	F	49	69	19	409	79	310	1: 160	80
3	F	44	58	15	1783	50	322	1: 640	403
4	F	51	74	17	839	40	250	1: 160	49
5	M	38	120	17	1730	98	203	1: 320	33
6	F	40	63	11	1203	45	154	1: 320	755
7	F	49	132	29	738	10	398	1: 160	23
8	F	17	49	9	1839	94	392	1: 640	732
9	F	29	100	29	1937	39	538	1: 640	537
10	F	32	73	23	2784	98	837	1: 320	304
11	F	25	84	20	1783	128	98	1: 320	893
12	F	21	13	26	1435	234	345	1: 320	374

13	F	45	134	14	849	135	245	nd	256
14	F	24	16	24	2543	124	245	1: 320	298
15	F	57	44	67	1145	83	245	nd	307.5
16	F	43	145	45	1454	145	244	1: 160	12
17	F	44	45	34	1545	345	464	1: 320	246
18	F	35	45	67	1974	273	385	nd	41
19	M	26	54	15	1395	133	156	1: 320	367
20	F	34	43	17	1833	48	493	1: 640	837
21	F	45	84	57	1695	84	573	1: 640	596
22	F	52	83	23	1637	173	337	1: 320	133
23	M	47	85	39	1355	345	453	1: 320	354
24	F	56	34	45	778	156	243	1: 640	567
25	F	38	63	78	974	258	175	1: 160	40
26	F	62	73	45	853	356	262	1: 160	83
27	F	29	94	47	1467	456	384	1: 320	339
28	M	24	73	39	1837	85	263	1: 640	639

29	F	53	37	34	849	273	252	1: 320	283
30	F	32	72	30	836	29	473	1: 160	103
31	F	19	39	53	1494	93	837	1: 640	473
32	M	35	130	39	1438	120	372	nd	123
33	F	44	173	12	1363	84	463	1: 320	652
34	F	63	83	32	735	74	183	1: 160	58
35	F	39	36	19	1523	25	483	1: 640	634
36	F	48	74	10	893	49	273	1: 160	53
37	F	20	48	12	1837	89	253	1: 320	82
38	F	52	66	15	1635	54	183	1: 320	472

nd: not detected

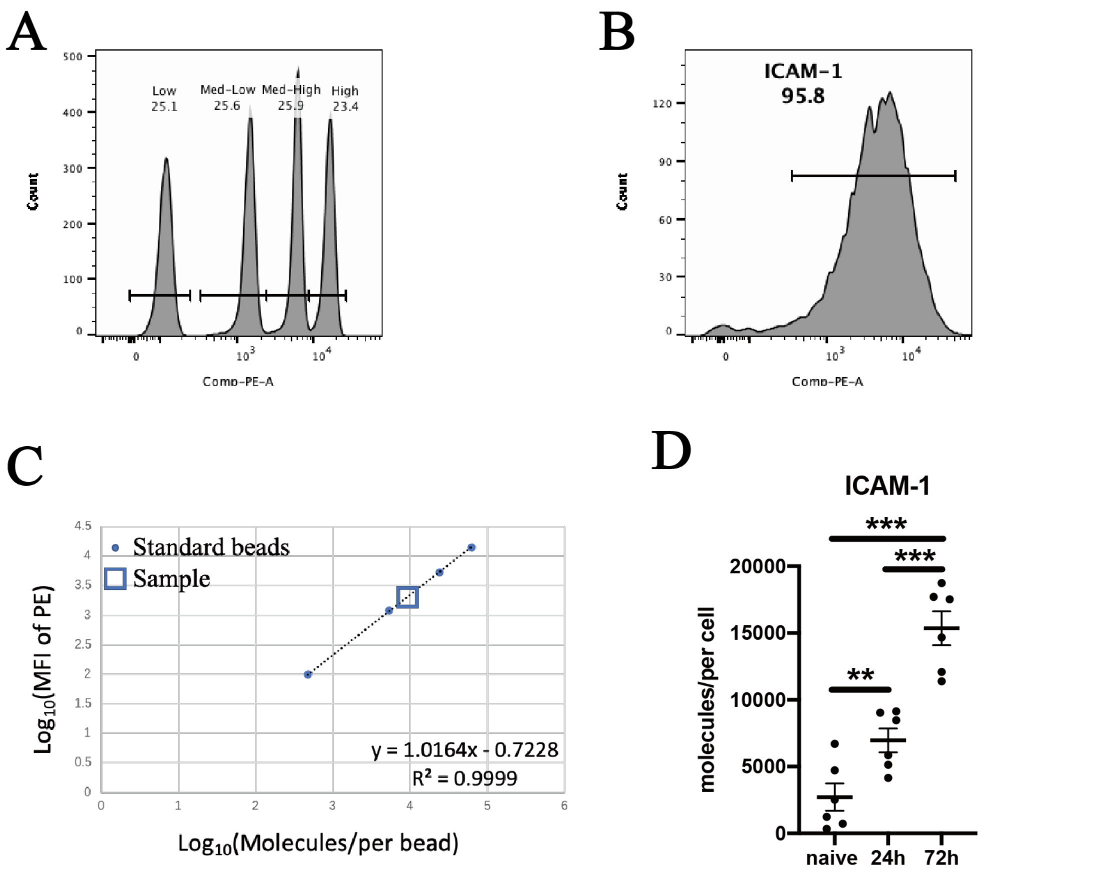

Figure S1. Determination of molecular density of ICAM-1 on CD4⁺ T cells, Related to Figure 3

(A) The standard calibration beads were analyzed by flow cytometry. (B) $CD4^+$ T cells incubated with PE-labeled primary anti-ICAM-1 mAb were determined by flow cytometry. (C) A calibration curve of PE molecules/per bead (provided by the manufacturer) vs mean fluorescence intensity PE-A was plotted based on data of four standard beads (*filled circles*). The site density of ICAM-1 on $CD4^+$ T cells was calculated by comparing the fluorescence intensity of the sample (*open square*) with the calibration curve. (D) The molecular density of ICAM-1 on stimulated $CD4^+$ T cells at different time point. The data were representative of three independent experiments. Data were represented as mean ± SD. The Kruskal-Wallis test with subsequent Duuns multiple-comparison test was used. **: $p < 0.01$; ***: $p < 0.001$.

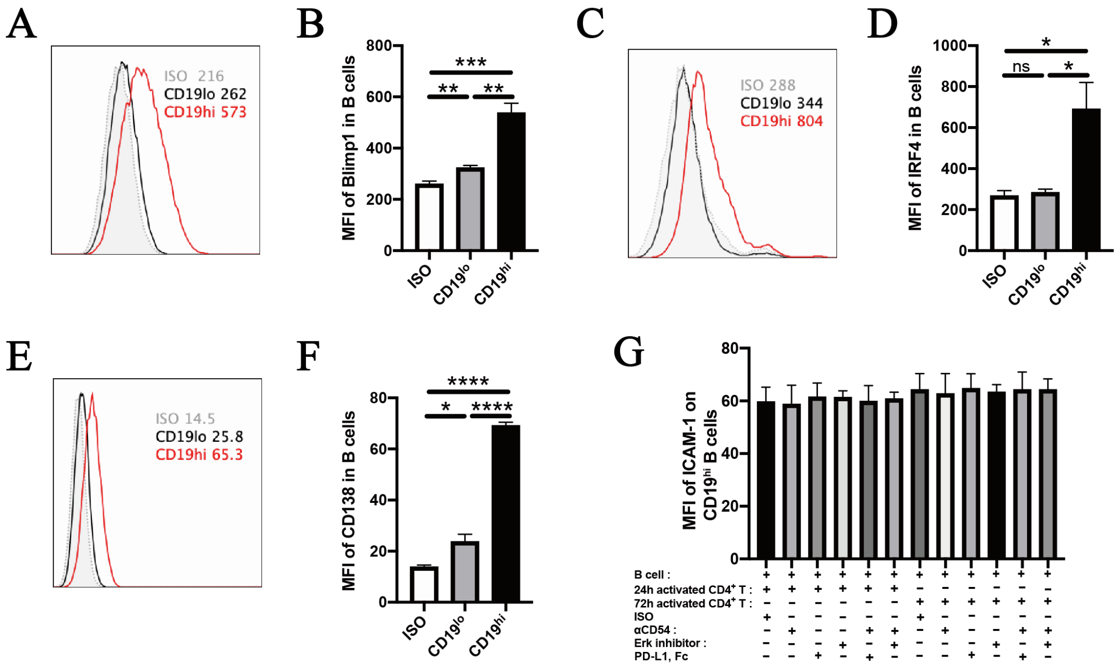

Figure S2. Blimp1, IRF4, CD138 and ICAM-1 expression on B cells after T-B co-culture, Related to Figure 1

Blimp1 (A-B), IRF4 (C-D) and CD138 (E-F) expression patterns were determined on $CD19^{lo}$ and $CD19^{hi}$ B cells after $CD4^{+}$T-B cell cocultures. We also determined ICAM-1 expression levels on $CD19^{hi}$ B cells (G) after freshly isolated B cells were cocultured with 72 hrs-activated $CD4^{+}$T cell in the presence of anti-ICAM-1 blocking antibody or the Erk1/2 inhibitor for 12 days. The data were representative of at least three independent experiments. Data was represented as mean ± SD. The Kruskal-Wallis test with subsequent Duuns multiple-comparison test was used. *: $p < 0.05$; **: $p < 0.01$; ***: $p < 0.001$; ****: $P < 0.0001$.

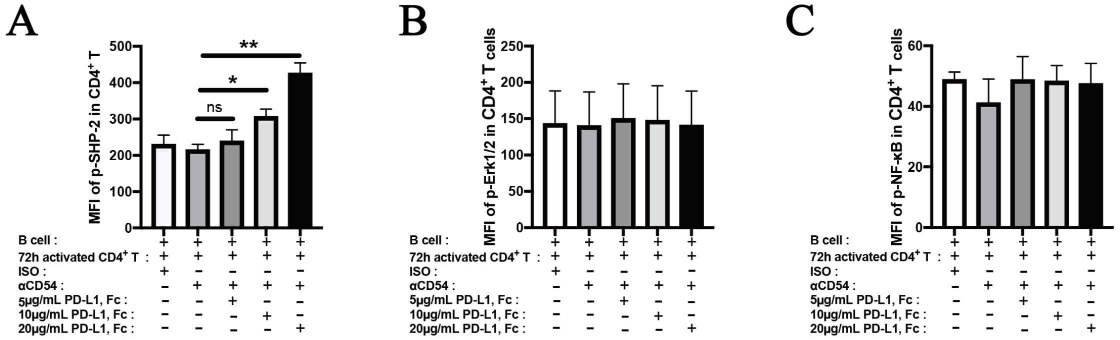

Figure S3. Activation of signaling molecules in CD4⁺ T cells after T-B co-culture, Related to Figure 6

(A-C) The MFI of phosphor-SHP-2 (Y542) (A), phosphor-Erk1/2 (pT202/pY204) (B) and phosphor-p65 (pS529) (C) in $CD4^+$ T cells after 72 hrs-activated $CD4^+$ T-B co-cultures with the addition of anti-ICAM-1 blocking antibody and PD-L1-Fc proteins at different final concentration (5, 10, 20 μg/mL) for 12 days. The data were representative of at least three independent experiments. Data was represented as mean ± SD. The Kruskal-Wallis test with subsequent Duuns multiple-comparison test was used. *: $p < 0.05$; **: $p < 0.01$.
